# RTCpredictor: identification of read-through chimeric RNAs from RNA sequencing data

**DOI:** 10.1093/bib/bbae251

**Published:** 2024-05-25

**Authors:** Sandeep Singh, Xinrui Shi, Samuel Haddox, Justin Elfman, Syed Basil Ahmad, Sarah Lynch, Tommy Manley, Claire Piczak, Christopher Phung, Yunan Sun, Aadi Sharma, Hui Li

**Affiliations:** Department of Pathology, School of Medicine, University of Virginia, Charlottesville, VA 22908, United States; Department of Pathology, School of Medicine, University of Virginia, Charlottesville, VA 22908, United States; Department of Biochemistry and Molecular Genetics, School of Medicine, University of Virginia, Charlottesville, VA 22908, United States; Department of Biochemistry and Molecular Genetics, School of Medicine, University of Virginia, Charlottesville, VA 22908, United States; Department of Biochemistry and Molecular Genetics, School of Medicine, University of Virginia, Charlottesville, VA 22908, United States; Department of Pathology, School of Medicine, University of Virginia, Charlottesville, VA 22908, United States; Department of Pathology, School of Medicine, University of Virginia, Charlottesville, VA 22908, United States; Department of Pathology, School of Medicine, University of Virginia, Charlottesville, VA 22908, United States; Department of Pathology, School of Medicine, University of Virginia, Charlottesville, VA 22908, United States; Department of Pathology, School of Medicine, University of Virginia, Charlottesville, VA 22908, United States; Department of Pathology, School of Medicine, University of Virginia, Charlottesville, VA 22908, United States; Department of Pathology, School of Medicine, University of Virginia, Charlottesville, VA 22908, United States; Department of Pathology, School of Medicine, University of Virginia, Charlottesville, VA 22908, United States; Department of Biochemistry and Molecular Genetics, School of Medicine, University of Virginia, Charlottesville, VA 22908, United States

**Keywords:** RTCpredictor, chimeric RNA, read-through, RNA-Seq

## Abstract

Read-through chimeric RNAs are being recognized as a means to expand the functional transcriptome and contribute to cancer tumorigenesis when mis-regulated. However, current software tools often fail to predict them. We have developed RTCpredictor, utilizing a fast ripgrep tool to search for all possible exon-exon combinations of parental gene pairs. We also added exonic variants allowing searches containing common SNPs. To our knowledge, it is the first read-through chimeric RNA specific prediction method that also provides breakpoint coordinates. Compared with 10 other popular tools, RTCpredictor achieved high sensitivity on a simulated and three real datasets. In addition, RTCpredictor has less memory requirements and faster execution time, making it ideal for applying on large datasets.

## Introduction

The identification of chimeric RNAs generated by intergenic splicing has opened up a new paradigm of transcriptional diversity wherein events are being discovered and experimentally validated in numerous studies [1–8]. Among them, a subset of chimeric RNAs have been identified as diagnostic markers and/or therapeutic targets in cancer patients [9–11], while the potential of many others is currently being explored [8, 12–14]. Chimeric RNAs have also been extensively reported in normal healthy tissues and cells [15–17], thereby challenging the traditional view of them being unique to neoplasia. A plethora of databases hosting chimeric RNAs have been developed and extensive annotations have provided insight about their functional relevance [3, 18–21]. Based on the coordinates from where the break points occur, chimeric RNAs can be grouped into three different types: (i) inter-chromosomal, where 5′ and 3′ parental genes of the chimeric RNA are located on different chromosomes; (ii) intra-chromosomal, where 5′ and 3′ parental genes are on the same chromosome, but are not next to each other on the same strand; and (iii) read-through, which involves two adjacent parental genes transcribing in the same direction.

Read-through chimeric RNAs, also known as products of cis-Splicing of Adjacent Genes (cis-SAGe), are generated when RNA polymerase extends transcription beyond a single gene to include a nearby gene on the same strand, and exons from these two parental genes are spliced together. The difference between read-throughs and intra-chromosomal chimeric RNAs originating from the same strand is that in the former case, the polymerization is extended by RNA polymerase passing through gene boundary, while the latter can be made from events like deletion or trans-splicing. Moreover, in the majority of the experimentally validated read-throughs from the Qin *et al*. study [22], the breakpoints of the parental genes are within 70 kb. Although read-through transcripts have been widely considered as a class of chimeric RNA [23], some categorize them as alternative splicing transcripts [13, 24, 25]. Traditionally, they were assumed to be biologically insignificant in cancer [26–28] because of the absence of genomic rearrangements. Therefore, they were often considered false positives and discarded by experimental biologists while screening for cancer-specific chimeric RNAs [29]. This assumption was manifested when computational biologists designed their prediction methods, filtering out these events using distance-based criteria [30]. However, a number of read-through chimeric RNAs with clear clinical and biological relevance are recently reported. Rickman *et al*. [31] identified chimeric RNA *SLC45A3-ELK4* in prostate cancer, demonstrating its biomarker capabilities, and Zhang *et al*. [32] classified it as a cis-SAGe event by elucidating the mechanism of formation. Further, Zhang *et al*. correlated this read-through chimera with Gleason score which is not seen with either parental gene transcript [32]. Varley *et al*. [33] screened 168 breast samples to identify and experimentally validate *SCNN1A-TNFRSF1A* and *CTSD-IFITM10* read-throughs present in breast cancer cell lines and primary tumors but not detected in normal tissue. By performing analysis of deep RNA-Seq in prostate cancer and reference samples, Nacu *et al*. [34] identified *MSMB-NCOA4* read-through that may play some functional roles in cancer. Recently, we data mined multiple cancer types from TCGA to explore the landscape of chimeric RNAs in cancer. We identified and experimentally validated several read-through chimeric RNAs (*LHX6-NDUFA8* in cervical cancer [35], *D2HGDH-GAL3ST2* in prostate cancer [36], *BCL2L2-PABPN1* in bladder cancer [37] and *RRM2-C2orf48* in colorectal cancer [38]) and have shown their potential to be used as diagnostic markers and/or therapeutic targets. Impressively, same clinical correlation was often not seen with the parental gene transcripts, and sometimes even opposite trend was observed as in the case of *RRM2-C2orf48* [38]. In addition, several papers have reported physiological read-through chimeric RNAs that are necessary for normal cell/tissue function [16, 39–41]. These data provide evidence that the read-through chimeric RNAs can have significant physiological and pathological roles.

It is evident that the inventory of read-through chimeric RNAs is far from saturation. There is a current need for computational methods to detect read-through chimeras. Currently available tools for chimeric RNA prediction are not sensitive enough to predict read-throughs. This is evident from our recent benchmark study which demonstrated that 16 chimeric RNA prediction methods lacked sensitivity to predict read-through chimeric RNAs [30]. Moreover, many software methods purposely discard read-through chimeric RNA events [30]. In this study, we describe Read-Through Chimeric RNA Predictor (RTCpredictor), a method designed specifically for the identification of read-through chimeric RNAs. We show comparison of RTCpredictor with ten prediction methods that had at least 5% sensitivity on read-throughs, based on our recent benchmark study. The methods are (i) JAFFA [24], (ii) SOAPfuse [42], (iii) EricScript [43], (iv) FusionCatcher [44], (v) ChimPipe [45], (vi) pizzly [46], (vii) InFusion [47], (viii) Arriba [27], (ix) FuSeq [48] and (x) ChimeraScan [49]. A short description of some of these methods is described elsewhere [50]. Briefly, except FuSeq, which uses k-mer based mapping, all other methods align the input reads either to the reference genome (Arriba) or transcriptome (JAFFA, EricScript, FusionCatcher) or both (SOAPfuse, ChimPipe, InFusion, ChimeraScan). It is followed by the identification of chimeric RNA supporting reads (split and spanning reads) and creation of an initial list of potential chimeric RNAs. Different types of filtering steps are implemented such as discarding paralog, pseudogene, tandem repeats, and the number of split and spanning reads to reduce false positive events. Methods such as JAFFA, Arriba and FuSeq either discard or give low priority to read-through chimeric RNAs. Most of these methods work on paired-end short reads, while FusionCatcher and JAFFA also support single-end reads. On simulated and three real datasets, RTCpredictor has displayed superior performance when predicting read-through chimeric RNAs. Our method is highly sensitive, fast and scalable in high-throughput settings.

## Materials and methods

### RTCpredictor algorithm

**Figure 1:**
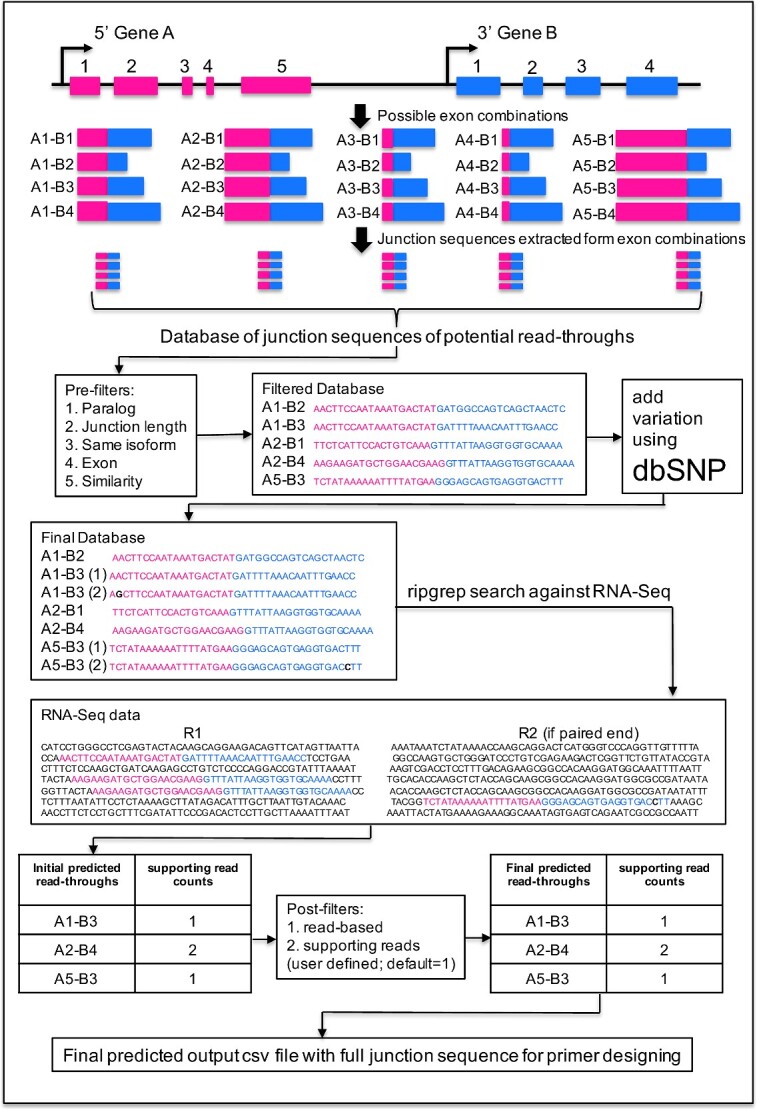
Flowchart of the RTCpredictor method. Exemplary genes A and B are located on the same strand with five and four exons, respectively. There are 20 possible exon combinations with the distance between breakpoints is ≤70 kb, which is used to generate a database of junction sequences of potential read-throughs. Pre-filters are applied to discard potential false positive events. dbSNP is used to map and add common variants on junction sequences to generate the final database. This final database is used to search against RNA-Seq input data using ripgrep for the initial predicted read-throughs. Post-filters are applied to exclude additional potential false positive events to generate the final list of predicted read-throughs. The annotations including genomic coordinates and junction sequences are included in the output file, which will help the users to design primers for their experimental validation.

We designed the algorithm to conduct an exhaustive search of junction sequences obtained from all possible exon combinations of neighboring genes against RNA-Seq data. We consider potential read-throughs as events where both 5′ and 3′ parental genes are transcribed in the same direction and the distance between 5′ and 3′ breakpoints is ≤70 kb, consistent with the criteria established in our previous benchmark study [30]. We extracted 20-bp sequences from each side of the breakpoints and combined them to form a 40-bp junction sequence. Using hg19 genome and Ensembl version 75 annotation file, we extracted 4 967 029 exon combinations representing 9 781 826 unique sequences (junction sequences and their reverse complements) from 110 877 gene pairs with distances within ≤70 kb (Figure 1). Using ripgrep string search software (https://github.com/BurntSushi/ripgrep), these 9.78 million junction sequences, each corresponding to a unique read-through transcript, were searched against RNA-Seq data to identify read-through events. This approach does not require mapping RNA-Seq data to the reference genome, making the algorithm very fast and free of errors introduced at the alignment step.

### Filters to discard potential false positive events

We implemented several filters to identify and discard potential false positive events. The first five of these filters are implemented before the ripgrep search, i.e. they discard potentially false positive junction sequences (exon combinations) (Figure 1). The other two filters are implemented after the ripgrep search. The filters are as follows: (1) paralog filter: since paralog genes arise from gene duplication events, misalignment of the reads can produce artifact read-throughs. Therefore, if the 5′ and 3′ genes of the potential read-through are paralogs of each other, those combinations were discarded (9 303 622 junction sequences retained). The list of paralog genes were downloaded from Ensemble version 75. (2) Junction sequence length filter: if 5′ or 3′ exon length is <20 bp, then a junction sequence of 40 bp cannot be constructed, which is required for the successful running of RTCpredictor; therefore, those combinations were discarded (9 146 174 junction sequences retained). (3) Same isoform filter: these events arise from overlapping genes. A read matching to common exons of two different but overlapping genes appears to support a read-through but actually belongs to the same transcript of one gene. If 3′ exon of the junction sequence is also present in 5′ gene and it is next to the 5′ exon and vice versa, then it is discarded (9 002 938 junction sequences retained). (4) Exon filter: the junction sequence is discarded if it matches to any exon in the genome. A BLAST database of all exon sequences of the hg19 genome was constructed and we performed a BLAST search of junction sequences against the exon database. Sequence identity ≥90% and alignment length covering ≥90% of the junction sequence was used as criteria to discard them (8 999 838 junction sequences retained). this filter eliminates 3100 junction sequences, which would lead to false positive read-throughs with multiple supporting reads from RNAseq data. (5) Similarity filter: it avoids the representation of alternatively spliced isoforms of a single gene in the junction sequence database and prevents these events to be predicted as a read-through. If the 5′ exon is similar to any exon of the participating 3′ gene and vice versa, then these events were discarded. A BLAST search of the 5′ or 3′ exon sequence was performed against all exons of the 3′ or 5′ gene, respectively, and sequence identity ≥70% with an alignment length coverage of ≥70% was used to discard events (8 789 234 junction sequences retained). After these filters, a database file was constructed containing 8 789 234 junction sequences with breakpoint coordinates, to be used for ripgrep against RNA-Seq data. (6) Read-based filter: after the ripgrep search, supporting reads were calculated for each read-through. If any supporting read was found to support more than one read-through, then that read was discarded and the supporting read counts were recalculated. If the discarded read was the only read supporting any read-through, then that candidate read-through entry was discarded. (7) Number of supporting reads: this filter is an optional filter provided to the users to select read-throughs with a minimum number of supporting reads.

### Mapping variation in junction sequences

RTCpredictor searches for an exact match of junction sequences against RNA-Seq data, and therefore may miss read-throughs with variation within the junction sequence. To address this, we identified 9372 *single nucleotide polymorphisms* (SNPs) within junction sequences with common allele frequency (CAF) ≥10% and included additional junction sequences reflecting common variations in our database file. We downloaded SNP data from dbSNP (version b151) using the GRCh37p13 genome and used bedtools (version 2.27.1) [51] to intersect with our junction sequences.

To enhance the applicability of RTCpredictor across genomes of different organisms, we have also provided a script to construct the database files for the genome of interest and provided instructions on how to download the prerequisite input files to run the script. Further, the distance parameter between 5′ and 3′ breakpoints can be modified by the user, with 70 kb as the set default value.

### Datasets

To evaluate the performance of our prediction method, we have used a simulated dataset and three real datasets containing different numbers of previously validated read-throughs. The details of these datasets are provided below:

### ChimPipe-simulated dataset

This dataset consists of 250 chimeric RNAs out of which 50 are chimeras between the same neighboring directional genes. Briefly, the dataset was created using the ChimSim module of ChimPipe package [45] wherein 250 simulated chimeric RNAs were mixed with 102 149 Gencode (version 19) transcripts, including transcripts from the parental genes contributing to chimeric RNAs. Three paired-end libraries were created with read lengths of 101, 76 and 50 bp and an estimated total number of reads of 31, 42 and 64 million, respectively. To contrast our method with others on 36 simulated read-throughs, we excluded 14 out of 50 where the 5′ and 3′ breakpoint coordinate distances was ≥70 kb, consistent with the established criteria in our previous benchmark study [30]. These 36 chimeric RNAs along with their breakpoint coordinates are given in Supplementary Table S1.

### PE150-simulated dataset

This simulated data set was prepared using the same fasta input file used for the original ChimPipe-simulated dataset and contains all the same exact chimeric RNA transcripts. We used Rsubread package [52] for generating the fastq files. This simulated dataset was made with a 150-bp read length and 30 million read depth with TPM values at 2 or higher for each chimera.

### Qin *et al*. real dataset

This dataset consists of 62 chimeric RNAs containing 46 read-throughs. These read-throughs were identified from the LNCaP prostate cancer cell line and were experimentally validated by RT-PCR and Sanger sequencing [22]. Consistent with our previous benchmark study [30], we used two runs (SRR1657556 and SRR1657557 from SRA study ID SRP050061) and applied quality filtering with the NGS QC Toolkit [53], using default parameters to select high quality reads. The read length of both runs is 101 with an estimated total number of 62 and 58 million reads after filtering, respectively. As was done in the simulated dataset, we excluded two out of 46 read-throughs with distances between 5′ and 3′ breakpoint coordinates >70 kb. We further excluded nine read-throughs whose 5′ and 3′ breakpoint coordinates did not map to the end and start of the participating exons. Therefore, we evaluated the performance of our method and others on 35 real read-throughs. These 35 chimeric RNAs along with their breakpoint coordinates are given in Supplementary Table S2.

### High-confidence dataset

This dataset was created by selecting predicted read-throughs on the Qin *et al*. real dataset that were called by at least two prediction methods. It gives the opportunity to compare the performance of all the methods on a consensus set of chimeric RNAs. Prior to creating this high-confidence set, from each software method, we selected the read-through chimeric RNAs with 5′ and 3′ genes on the same chromosome and strand that had a distance between their breakpoints ≤70 kb. We also removed the chimeric RNAs having clone-based gene names starting with letters ‘RP’. We obtained a total of 289 events in this set (Supplementary Table S3).

### TCGA bladder dataset

This dataset contains six experimentally validated read-through chimeric RNAs that were identified from TCGA bladder cancer RNA-Seq data using EricScript software [37]. Each read-through was detected in multiple samples, so we ran all programs on three randomly selected samples to predict each read-through. If the software detected the read-through in any of the three samples, then we consider that the software successfully re-identified the read-through. The EricScript software was not compared on this dataset because it is the tool that was used to identify these six read-throughs in the TCGA bladder cancer study. The sample IDs corresponding to each read-through are provided in Supplementary Table S4.

### TCGA colorectal dataset

This dataset contains eleven experimentally validated read-through chimeric RNAs that were identified from TCGA colorectal cancer RNA-Seq data using EricScript software [38]. We used the above-described approach as in the TCGA bladder cancer dataset to randomly select three samples for each read-through and ran all the software methods (except EricScript). If the software detected the read-through in any of the three samples, then we consider this a success. The sample IDs corresponding to each read-through are provided in Supplementary Table S5.

### Performance parameters

We have used Sensitivity to calculate the performance of our prediction method and comparing with other methods. Sensitivity is the percentage of correctly predicted true positive events. It should be noted that PPV and F-measure are not well suited for comparing the performance of chimeric RNA prediction methods on real datasets. The exact number of true chimeric RNAs in real datasets is unknown, and the PPV and *F*-measure of a software is underestimated if it predicts an unknown but true chimeric RNA [30]. All previous benchmark studies have compared the performance of methods based on gene pairs. However, it is critical for downstream users to know the exact breakpoints. Therefore, in this study, we compared the performance of our method and others based on breakpoint coordinates at the isoform level. Prediction at the isoform level is more difficult and thus more stringent than predicting at the gene pair level.

### Computational time and memory calculation

To calculate the time and memory load required for running this prediction method, we used the ‘time’ command, which is a built-in command in the Linux operating system. We reported time in minutes and memory load in gigabytes (GB). Consistent with our previous benchmark study [30], we used the same high-performance computing system and SRR1657556 sample to test computational load for one-to-one comparison with other methods. Briefly, a single core on a single node having Intel Xeon processor (E5-2630 @2.40GHz) was used to run individual software methods.

### Experimental validation

RNA extracted from the LNCaP prostate cancer cell line was reverse transcribed via Verso cDNA Synthesis Kit (Thermo Scientific) [54]. Primer pairs were designed using Primer3 targeting the relative upstream and downstream exons of the junction sequence. The primer design followed these rules: (1) primer length is between 18 and 20 bp. (2) Primer Tm is between 57 °C and 63 °C. (3) Primer GC% is between 40 and 60%. (4) Poly-X of primer is no more than 3. (5) Primer targeting site is >50 bp away from the junction site, when possible. All primer sequences are listed in Supplementary Table S6. A Step One Plus Real-Time PCR System (Applied Biosystems) was used to perform SYBR Green-based qRT-PCR experiments. Amplified products were separated using agarose gel electrophoresis. Proper-sized product bands were purified using the PureLink Quick Gel Extraction Kit (Invitrogen) and submitted for Sanger sequencing (Genewiz). To validate candidate chimeras, PCR product sequences obtained from Sanger sequencing were aligned to the human genome using BLAT tool.

## Results

### Performance on the ChimPipe-simulated dataset

**Figure 2:**
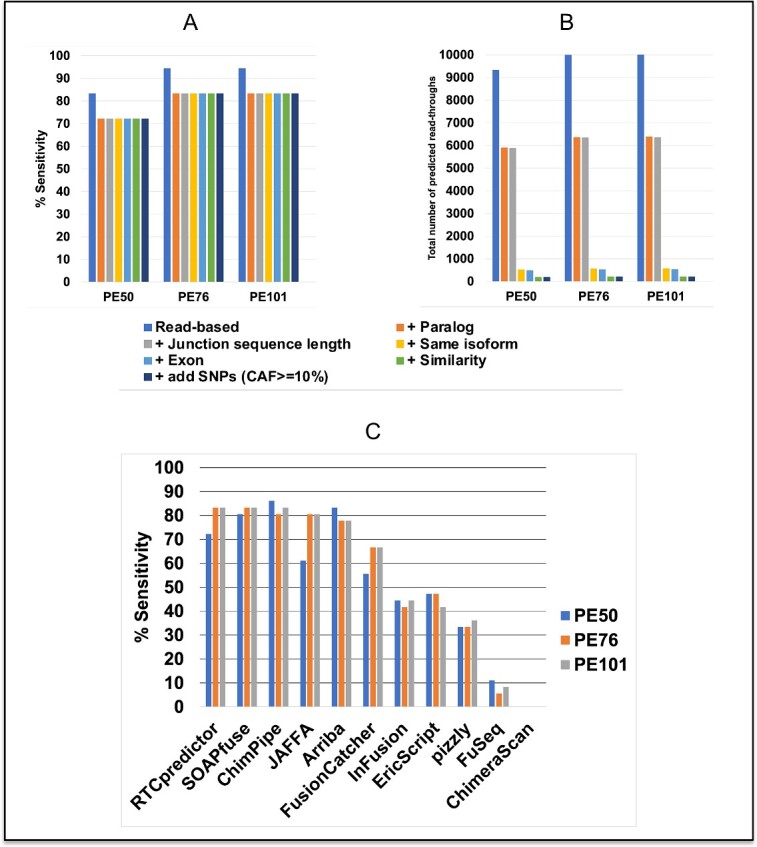
Performance of RTCpredictor and its comparison with other methods on the ChimPipe-simulated (PE50, PE76 and PE101) datasets. (**A**) % Sensitivity achieved by RTCpredictor and (**B**) total number of predicted read-throughs after sequential addition of different filters. (**C**) Comparison of % Sensitivity of RTCpredictor with other methods.

ChimPipe generated a simulated dataset, which contains a number of chimeric RNAs with a subset of 36 considered meeting the requirement of read-throughs. RTCpredictor achieved 94.4, 94.4 and 83.3% sensitivity on ChimPipe PE101, PE76 and PE50 datasets, respectively, when only the read-based filter was applied (Figure 2A and Supplementary Table S7). However, it also provided a lot of predictions (10 032, 9995 and 9322 on PE101, PE76 and PE50, respectively), that may be potential false positives. Although RTCpredictor predicted all 36 read-throughs on the PE101 and PE76 datasets, *CT45A2-CT45A3* and *HBA2-HBA1* were discarded by the read-based filter and therefore sensitivity dropped to 94.4%. In the PE50 dataset, four additional read-throughs (*AIMP2-ANKRD61*, *ALOX15B-AC129492.6*, *ZFYVE28-MXD4* and *ZNF837-ZNF497*) were overlooked by RTCpredictor due to short read length (50 bp) of the PE50 dataset because the junction sequences searched by RTCpredictor are 40 bp in length. After adding the paralog filter, four additional read-throughs were discarded and the sensitivity dropped to 83.3, 83.3 and 72.2% on PE101, PE76 and PE50 datasets, respectively. Importantly, applying four more filters (Junction sequence length filter, Same isoform filter, Exon filter and Similarity filter) did not affect sensitivity. However, it did drastically reduce the total number of predictions (213, 210 and 191 on PE101, PE76 and PE50, respectively) by discarding additional potential false positives (Figure 2B and Supplementary Table S7). The total number of predicted read-throughs remained the same, even with the addition of common SNP variations (CAF ≥ 10%).

We also compared the performance of RTCpredictor with other chimeric RNA prediction methods using the ChimPipe-simulated dataset. Consistent with our previous benchmark study [30], we chose the following 10 most popular chimeric RNA prediction methods: (i) JAFFA [24], (ii) SOAPfuse [42], (iii) EricScript [43], (iv) FusionCatcher [44], (v) ChimPipe [45], (vi) pizzly [46], (vii) InFusion [47], (viii) Arriba [27, 28], (ix) FuSeq [48] and (x) ChimeraScan [49]. We did not include INTEGRATE, MapSplice, STARChip, STAR-Fusion and TopHat-Fusion methods because their sensitivity on all read-through datasets was less than 5% [30]. We also excluded the ChimeRScope or ARTDeco methods because they do not provide the breakpoint coordinates of predicted chimeric RNAs. We adjusted the parameters of all methods, when possible, to include read-through chimeric RNAs in their predictions (Supplementary Table S8). We downloaded the output files of all methods benchmarked in our previous study to calculate their performance [30].

On the ChimPipe PE101 dataset, our RTCpredictor method achieved the highest sensitivity along with leading performers SOAPfuse and ChimPipe (83.3%), while JAFFA (80.6%) and Arriba (77.8%) performed second and third best, respectively (Figure 2C and Supplementary Table S9). Similarly, on the ChimPipe PE76 dataset, RTCpredictor along with SOAPfuse were the top performers (83.3%), while ChimPipe and JAFFA (80.6%) performed second best and Arriba (77.8%) was third best (Figure 2C). On the ChimPipe PE50 dataset, RTCpredictor dropped to the fourth, which is anticipated because RTCpredictor searches junction sequences of 40 bp in length against short reads of 50 bp.

### Performance on PE150-simulated dataset

This dataset is just like the ChimPipe-simulated dataset with the only difference that it has bigger read length of 150 bp. On this dataset, we were able to test following software methods: RTCpredictor, EricScript, FuSeq, pizzly, Arriba, FusionCatcher, InFusion, JAFFA and SOAPfuse. ChimPipe and ChimeraScan failed during the process. On this dataset, among these methods, RTCpredictor achieved the highest sensitivity (83.3%), followed by JAFFA (80.6%) and SOAPfuse (75%) (Supplementary Table S9). The sensitivities achieved by FusionCatcher, Arriba, InFusion, EricScript, pizzly and FuSeq are 66.7, 52.8, 44.4, 38.9, 36.1 and 19.4%, respectively. When we compared the performance of all the methods without filtering out any chimeric RNA from the dataset, the best sensitivity was achieved by JAFFA (84%), followed by SOAPfuse (74%), FusionCatcher (64%) and RTCpredictor (60%) (Supplementary Table S10).

### Performance on Qin *et al.*, TCGA bladder and TCGA colorectal cancer real datasets

**Figure 3:**
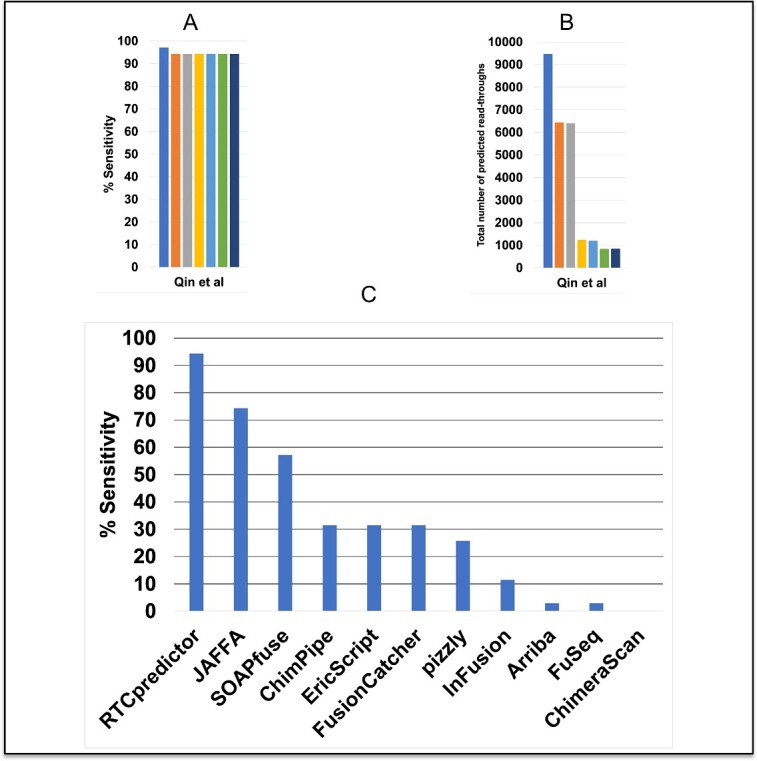
Performance of RTCpredictor and its comparison with other methods on the Qin *et al*. real dataset. (**A**) % Sensitivity achieved by RTCpredictor, (**B**) total number of predicted read-throughs, after sequential addition of different filters, and (**C**) comparison of % Sensitivity of RTCpredictor with other methods.

Qin *et al*. have experimentally validated 62 chimeric RNAs, among which 46 are considered read-throughs. RTCpredictor achieved 97.1% sensitivity on the Qin *et al.* real dataset, missing only one read-through (*BRCA1-VAT1*). However, a total of 9474 read-throughs were predicted. The paralog filter discarded an additional read-through (*AKAP8L-AKAP8*), dropping the sensitivity to 94.3%. After applying four additional filters, the sensitivity remained the same but the total number of predictions decreased drastically to 841 read-throughs. After the addition of common SNP variations (CAF ≥ 10%), nine more read-throughs were predicted, bringing the total to 850 (Figure 3A, B and Supplementary Table S7).

On the TCGA bladder cancer dataset, RTCpredictor re-identified three out of six known read-throughs (*ACKR2-KRBOX1, CHCHD10-VPREB3 and SLC2A11-MIF*) (Supplementary Table S11). On the TCGA colorectal cancer dataset, RTCpredictor re-identified five out of eleven known read-throughs (*CTSD-IFITM10, GCSH-C16orf46, METTL21B-TSFM, SLC2A11-MIF and TRIM2-MND1*) (Supplementary Table S12).

We also compared the performance of RTCpredictor with other methods on these real datasets. On the Qin *et al.* real dataset, RTCpredictor achieved significantly higher sensitivity (20% more) than the second-best method, JAFFA. RTCpredictor (94.3%) was the top performer while JAFFA (74.3%) and SOAPfuse (57.1%) performed second and third, respectively (Figure 3C and Supplementary Table S9). When we compared the performance of all the methods without filtering out any chimeric RNA from the Qin *et al*. real dataset, RTCpredictor still remained best sensitive method followed by JAFFA and SOAPfuse (Supplementary Table S10).

On the TCGA bladder cancer dataset, RTCpredictor re-identified three out of six known read-throughs (50% sensitivity) followed by SOAPfuse, FusionCatcher and ChimPipe that re-identified two read-throughs (33.3%) each, while FuSeq and pizzly re-identified a single read-through (16.7%). JAFFA, Arriba, ChimeraScan and InFusion did not identify any read-throughs (0%) (Supplementary Table S11).

On the TCGA colorectal cancer dataset, RTCpredictor re-identified six out of eleven known read-throughs (54.5% sensitivity), followed by SOAPfuse and ChimPipe, which re-identified three read-throughs each (27.3%). JAFFA and FuSeq re-identified two read-throughs (18.2%) and FusionCatcher and InFusion re-identified a single read-through (9.1%). Arriba, ChimeraScan and pizzly were not able to identify any read-through (0%) (Supplementary Table S12).

### RTCpredictor uncovered actual read-throughs in the ChimPipe-simulated dataset

The authors of the ChimPipe dataset simulated only 250 chimeric RNAs, out of which 36 matches the definition of read-throughs. However, RTCpredictor predicted 213, 210 and 191 read-throughs on PE101, PE76 and PE50, respectively. Moreover, all 210 read-throughs from PE76 were also predicted in PE101, and all 191 read-throughs from PE50 were also predicted in PE76. Sequences from the existing transcriptome were included in the ChimPipe-simulated dataset by the authors, and the transcriptome itself contains transcripts annotated as ‘read-throughs’. We therefore reasoned that some of our predictions may match already annotated read-throughs from the transcriptome. In the Ensembl version 75 annotation from the hg19 genome, 371 transcripts from 110 genes are annotated as read-throughs. We performed a BLAST search, with ≥90% sequence identity and ≥ 90% alignment length, of predicted read-through sequences (constructed by combining 5′ and 3′ exon partners) against these 371 transcript sequences to determine if any of our predicted read-throughs match with annotated read-throughs. There were 27, 27 and 23 predicted read-throughs from PE101, PE76 and PE50, respectively, that matched with annotated read-throughs from the transcriptome. Therefore, these are additional to the list of read-throughs which were simulated in the ChimPipe data. It remains possible that other predicted read-throughs may be also actual read-throughs of the transcriptome that have yet to be annotated.

### Experimental validation of additional read-throughs on the Qin *et al*. dataset

**Figure 4:**
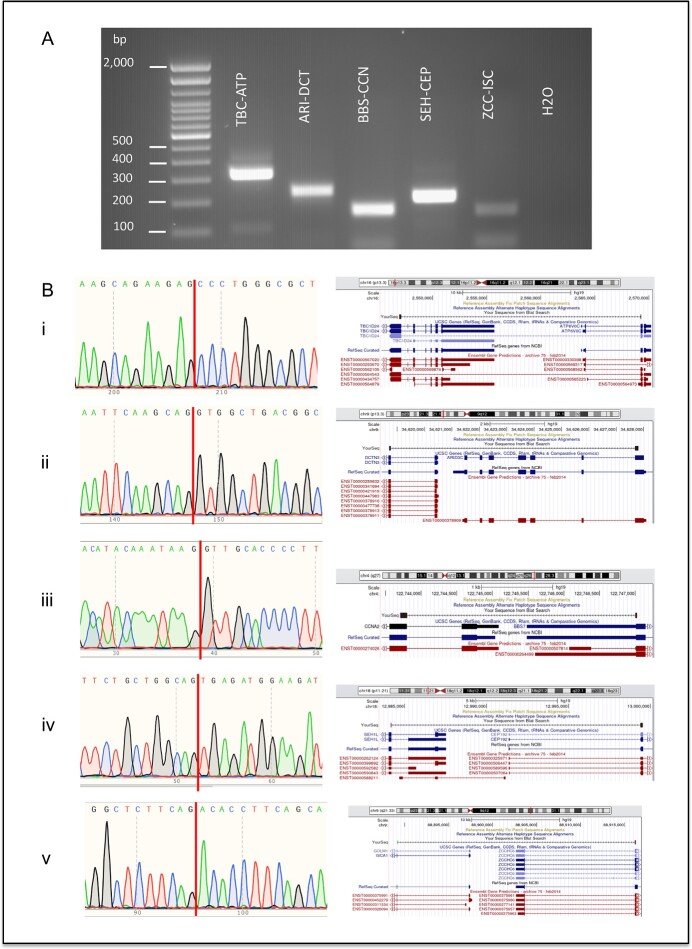
Experimental validation results of a subset of predicted read-through chimeric RNAs from the extended Qin *et al*. real dataset. (**A**) Experimental validation using RT-PCR. Bands at the bottom of the gel are primer dimers. (**B**) Junction sequences obtained from Sanger sequencing validation and visualization on UCSC genome browser for the following exemplary read-through chimeric RNAs (i) *TBC1D24-ATP6V0C*, (ii) *ARID3C-DCTN3*, (iii) *BBS7-CCNA2*, (iv) *SEH1L-CEP192* and (v) *ZCCHC6-ISCA1.*

For real dataset, PPV evaluation is not valid, in that the total true positive events are not known. We believe that the Qin *et al*. real dataset may have more than 46 true read-through events. To be fair to all software tools in this dataset, we randomly selected up to 30 read-through chimeric RNAs predicted by each software method for experimental validation. We followed these steps to select candidates for each software method: (i) we selected chimeric RNAs with 5′ and 3′ genes on the same chromosome and strand that had a distance between their breakpoints ≤70 kb. (ii) We removed 46 and 8 gene pairs from this list that were experimentally validated in the Qin *et al*. study and other individual studies, respectively [15, 22, 37, 38]. (iii) We removed 40 known read-through gene pairs which we previously published in a list of GTEx chimeric RNAs that are present across all 53 human tissues. In the (ii) and (iii) filtering steps, we used gene pairs instead of their coordinates to filter out the events because this way in our validation step, we ensured to get more diverse novel experimentally validated read-throughs rather than selecting different isoforms of the same gene pairs whose some isoforms are already known to be experimentally validated. (iv) We removed read-throughs having clone-based gene names starting with letters ‘RP’. We selected up to 30 candidates from each software prediction. For software tools that predicted less than 30 candidates, we included all for experimental validation. After combining the selected lists from all software methods, we had 208 unique read-through chimeric RNAs. Finally, we performed experimental validation on these 208 candidate read-throughs using RT-PCR. For read-throughs which showed an RT-PCR gel band, we performed additional validation using Sanger sequencing to confirm that the sequence matched the junction sequence of the predicted read-through chimeric RNA. In the end, 112 out of 208 read-throughs were experimentally validated, with 96 read-throughs either failing to produce an RT-PCR band or failing in the Sanger sequencing validation step. We classify these additional 112 experimentally validated read-throughs as an extended Qin *et al.* real dataset. The list of 208 read-throughs along with their validation status is given in Supplementary Table S6. The distribution of read-throughs in each step of the selection criteria is given in Supplementary Table S13. We have included RT-PCR gel bands, Sanger sequence results and visualization on the UCSC genome browser for five experimentally validated read-through chimeric RNAs from the extended Qin *et al.* real dataset that were predicted by RTCpredictor in Figure 4.

### Performance comparison of all the methods on the extended Qin *et al.* real dataset

With the addition of 112 experimentally validated read-throughs, the Qin *et al.* dataset now hosts the largest known count of read-through chimeric RNAs among various studies. We thus calculated the performance of all methods on this newly validated set. RTCpredictor (58%) achieved the highest sensitivity followed by JAFFA (42%) and pizzly (22.3%), while Arriba (0.9%), InFusion (0.9%) and ChimPipe (6.3%) performed poorly (Supplementary Table S14). After adding 35 read-throughs (from the original study) with these 112, the total number of experimentally validated read-throughs in the Qin *et al.* real dataset becomes 147. RTCpredictor achieved the highest sensitivity (66.7%) followed by JAFFA (49.7%) and pizzly (23.1%), while Arriba (1.4%) and InFusion (3.4%) performed poorly (Supplementary Table S15).

#### Performance on high-confidence dataset

We also compared the performance of all the methods on consensus predictions on the Qin *et al.* real dataset, those which are predicted by at least two software methods. Out of 289 read-throughs in this high-confidence set, RTCpredictor correctly identified 278, achieving highest sensitivity (96.2%), followed by JAFFA (82.7%) and pizzly (20.4%). In terms of the F1 score as well, RTCpredictor achieved highest score (0.6), followed by JAFFA (0.49) and pizzly (0.32). Next, we intersected this high-confidence set with 147 experimentally validated read-throughs from the extended Qin *et al.* real dataset and got 87 common read-throughs among them. We compared the performance of the methods on these 87 common read-throughs. Again, RTCpredictor achieved highest sensitivity (95.4%), followed by JAFFA (72.4%), SOAPfuse (33.3%) and pizzly (31%) (Supplementary Table S16).

#### Performance on PacBio single-molecule real-time sequencing data

Single-molecule real-time sequencing, developed by Pacific BioSciences (PacBio), offers much higher confidence in terms of transcript isoforms. Through a separate study, we had experimentally identified 18 read-throughs in human sperm samples (manuscript in preparation). Now that we have PacBio sequencing on the same RNA samples, we tested the performance of RTCpredictor on this long-read sequencing platform. Out of the 18 confirmed readthroughs, RTCprecdictor successfully identified 14. None of the other tools mentioned above works for the PacBio sequencing (JAFFA has a version of JAFFAL, which failed to generate prediction on the same dataset within 6 days).

### Computational requirements

**Figure 5:**
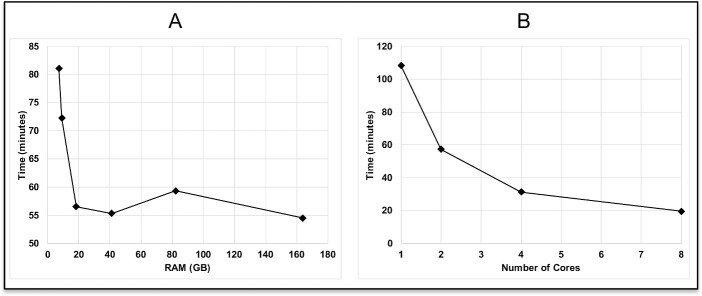
Computational requirements (time and memory) of RTCpredictor when (**A**) a single core is used and (**B**) multiple cores are used.

Using one core, RTCpredictor took ~55 min to complete the run for SRR1657556 but used a very high RAM of 164 GB (Figure 5A and Supplementary Table S17). This is due to the ripgrep software used by RTCpredictor processing all ~nine million junction sequences at once, thereby increasing RAM usage. Although research institutions equipped with high performance computing systems can afford high RAM, it may be challenging for researchers with limited resources. Therefore, we introduced a parameter in RTCpredictor that asks the user for the RAM (in GB) they can comfortably provide. If users provide less than 164-GB RAM, RTCpredictor splits junction sequences into multiple parts accordingly and processes each part one by one, thereby reducing RAM usage at the cost of running time (Figure 5A and Supplementary Table S17). The minimum RAM necessary for RTCpredictor is 5 GB per core, which is readily available even in present day laptops. Using one core and ~ 5 GB RAM, RTCpredictor took 109 min to complete (Figure 5B and Supplementary Table S17). RTCpredictor is significantly faster than the second-best sensitive method, JAFFA, which took 525 min to complete [30]. We have also tested RTCpredictor on dataset, SRR1657557, which contains about 58 million reads, as well as the same data but concatenated five times, which is about 290 million reads. We then compared the results from 5 GB RAM versus 164GB RAM. The results are identical, although 5-GB RAM took a longer time. To ramp up the speed of RTCpredictor, we also implemented multiprocessing options using the ParallelForkManager perl module, where users can use more than one core. With eight cores, RTCpredictor took <20 min to complete (Figure 5B and Supplementary Table S17).

## Discussion

Read-throughs as a class have been neglected in the quest to discover chimeric RNAs with biomarker or therapeutic potential. Their mechanism of formation does not involve chromosomal rearrangement, a classic feature of cancer, and therefore were discarded by many chimeric RNA prediction methods as false positives. Consequently, popular chimeric RNA prediction methods poorly predict the read-through class of chimeric RNAs, especially on real datasets, as demonstrated by our latest benchmark study [30]. In this study, we have developed the RTCpredictor method specifically for the prediction of read-through chimeric RNAs. We compared RTCpredictor with 10 other methods and found RTCpredictor achieved high sensitivity on both simulated and real datasets, with 20% higher sensitivity than the second-best method on the Qin *et al.* real dataset. Consistently, RTCpredictor also re-identified more known read-throughs than other methods on TCGA bladder and colorectal cancer real datasets.

To identify read-throughs, RTCpredictor uses ripgrep to search for ~nine million junction sequences of potential read-throughs against RNA-Seq data. Other chimeric RNA prediction methods use a mapping approach based on alignment to the genome or transcriptome, fetching spanning and split/junction reads and require at least one split/junction read to predict any chimeric RNA. However, as reported by Panagopoulos *et al.* [55], a simple grep utility (an in-built command in Linux OS) to search the *CIC-DUX4* chimeric RNA sequence outperformed three chimeric RNA prediction methods that use the mapping approach. Grep can be used to search few junction sequences, though it cannot handle ~nine million junction sequences. In contrast, a similar tool ‘agrep’ [56] does allow errors in the match, and has been used for *in silico* validation of chimeric RNAs [38, 57]. Although it is faster than grep, agrep also cannot handle millions of sequences. However, ripgrep can search for the millions of junction sequences efficiently. Like grep, ripgrep can only search for exact matches. Therefore, to accommodate for sequence variations among different individuals, we mapped SNP data onto the junction sequences and added the junction sequence variants in the searchable database.

We experimentally validated 112 read-throughs from a pool of 208 predicted read-throughs obtained from all software methods on the Qin *et al.* dataset. Here, RTCpredictor outperformed all other methods and identified the highest number of experimentally validated read-throughs out of 112. Adding the original 35 read-throughs, the total number of experimentally validated read-throughs increased to 147, which makes this dataset the biggest real dataset for read-through chimera. It will be a valuable resource for any future benchmarking study.

In any real dataset, the total number of true positive events is not known and there might be more true positive events than the predicted list. Therefore, performance parameters like PPV (fraction of true chimeric RNAs out of total predictions) and *F*-measure (balance of sensitivity and PPV) are not suitable for benchmarking chimeric RNA prediction methods because a tool is penalized if it correctly predicts unknown but true chimeric RNAs. In the latest benchmark study, we observed a similar occurrence on the Edgren dataset where there were originally 27 experimentally validated chimeric RNAs, which later increased to 99. This resulted in changes of the performance of the methods and thereby their rankings [24, 29, 30, 58].

In terms of computational requirements, RTCpredictor (~5-GB RAM) is 4.8 times faster than the second-best sensitive method JAFFA (~5-GB RAM), and 6.7 times faster than the third-best sensitive method SOAPfuse (~6-GB RAM) when using a single core and on the same sample [30].

In summary, our newly developed tool RTCpredcitor has the following advantages and some limitations. Advantages of RTCpredictor include the following: (i) high sensitivity, (ii) fast run time with a multiprocessing option, (iii) very easy installation, (iv) can be run on desktop/laptop with 5 GB RAM, (v) can be used for long-read data (PacBio platform) or short-read data having minimum read length of 50 and with any read-depths, on any sequencing platforms (illumina, Roche 454, Ion torrent, etc.), (vi) can be used to predict read-throughs from RNA-Seq data of any organism of interest, (vii) compatible with fastq file formats in compressed (^*^.gz) or uncompressed form, and (viii) provides full sequences of participating exons for primer designs in the experimental validation step. Limitations of RTCpredictor are as follows: (i) it cannot predict read-throughs whose breakpoints do not map to the end/start of exons or if the distance between the breakpoints of the parental genes is >70 kb, and (ii) it may miss read-throughs if long-reads from the Oxford Nanopore platform are used because the sequences may have a high error rate. A limitation of this study is as follows: for the benchmarking purpose, this study includes only those software methods which provide breakpoint coordinates of predicted read-throughs. Therefore, this study did not include methods such as ARTDeco [59], which is designed for the detection of read-through, but does not provide breakpoint coordinates of parental genes. These types of methods can be a useful resource to gain a list of read-through gene-pairs whose breakpoint coordinates can be obtained by performing an additional alignment step.

**Key Points**

- RTCpredictor is the first tool specifically designed for read-through chimeric RNAs.
- RTCpredictor outperforms most other popular tools in terms of sensitivity on simulated and real datasets.
- RTCpredictor works on short- and long-read RNA-sequencing data.
- RTCpredictor is fast and requires less memory.

## Supplementary Material

30mar24_SupplementaryTables_bbae251

## Data Availability

All data generated or analyzed during this study is included in this published article. Standalone version of RTCpredictor is available at https://github.com/sandybioteck/RTCpredictor
